# Predictors of recurrence based on intravascular ultrasound findings after Eluvia placement in symptomatic peripheral arterial disease: A retrospective study

**DOI:** 10.1002/hsr2.1481

**Published:** 2023-08-03

**Authors:** Takehiro Yamada, Takahiro Tokuda, Naoki Yoshioka, Akio Koyama, Ryusuke Nishikawa, Kiyotaka Shimamura, Takuma Aoyama

**Affiliations:** ^1^ Division of Cardiology Central Japan International Medical Center Gifu Japan; ^2^ Division of Cardiology Nagoya Heart Center Nagoya Japan; ^3^ Division of Cardiology Ogaki Municipal Hospital Ogaki Japan; ^4^ Division of Vascular surgery Toyota Memorial Hospital Toyota Japan; ^5^ Division of Cardiology Kyoto University Hospital Kyoto Japan; ^6^ Division of Cardiology Shizuoka General Hospital Shizuoka Japan; ^7^ Division of Molecular Pathology Shinshu University of Medicine Matsumoto Japan

**Keywords:** endovascular therapy, lower extremity artery disease, polymer‐coated paclitaxel‐eluting stent, predictor

## Abstract

**Background and Aims:**

Polymer‐coated drug‐eluting stents (Eluvia) have shown favorable clinical outcomes in real‐world registries. There are no reports on recurrent predictors after Eluvia placement based on intravascular ultrasound (IVUS) findings.

**Methods:**

We analyzed clinical data from the ASIGARU PAD registry, a retrospective, multicenter, observational study that enrolled patients who underwent endovascular therapy for superficial femoral and proximal popliteal arteries lesions using Eluvia or drug‐coated balloon. The primary outcome was the identification of recurrent predictors, including IVUS parameters at 12 months. The rate of target lesion recurrence was also assessed.

**Results:**

IVUS images were obtained in 54 of 65 cases. Seven recurrent cases (13.0%) were observed within 12 months. The random survival forest method presented eight predictive variables of recurrence: Clinical Frailty Scale (CFS), distal stent edge area, distal plaque burden, age, sex, distal external elastic membrane (EEM) area, minimum stent area (MSA), and distal lumen area. Furthermore, the partial dependence plot showed that frailty (CFS ≥ 6), smaller distal stent edge area, higher and lower distal plaque burden, older and younger age, female sex, smaller distal EEM area, smaller MSA, and smaller and larger distal lumen area predicted recurrence after Eluvia placement within 12 months.

**Conclusion:**

CFS, distal stent edge area, distal plaque burden, age, sex, distal EEM area, MSA, and distal lumen area were significant recurrent predictors after Eluvia placement.

## Key points

Eluvia have shown favorable clinical outcomes in real‐world registries. Predictors of recurrence after Eluvia placement based on intravascular ultrasound (IVUS) findings are unclear. Clinical Frailty Scale, age, sex, and distal IVUS findings were significant predictors of restenosis.

## 1 INTRODUCTION

The 2017 European Society of Cardiology guidelines indicate that plain old balloon angioplasty (POBA) and bare‐metal stent (BMS) implantation are the current standard endovascular revascularization strategies for femoropopliteal lesions.^1^ However, the recurrence rates after POBA and BMS placement were reported to be 43%–68% and 24%–36% at 1 year, respectively, and this high rate is an important issue.^2^, ^3^, ^4^, ^5^, ^6^ Although a polymer‐free drug‐eluting stent (DES), launched in 2012, presented a good primary patency rate of 83.1% at 1 year and 66.4% at 5 years in a pilot randomized controlled trial,^6^, ^7^ the ZEPHYR registry revealed insufficient primary patency of 50% at 1 year in patients with severe lesion characteristics.^8^ There have been significant recent advances in drug technology. The polymer‐coated DES (Eluvia, Boston Scientific) showed significantly improved primary patency compared with polymer‐free DES.^9^ Eluvia also yielded favorable patency rates in real‐world registries with severe lesion characteristics, confirming its usefulness in a clinical setting.^10^, ^11^, ^12^ Although several predictors of recurrence after Eluvia placement have been mentioned in real‐world registries, the evidence is still limited.^10^, ^12^ Moreover, there are no reports on predictors of recurrence after Eluvia placement based on intravascular ultrasound (IVUS) findings.

## 2 METHODS

### 2.1 Participants

**Figure 1:**
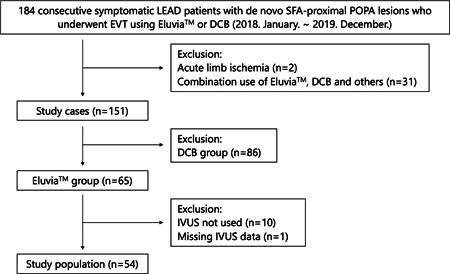
Flow diagram of the study population selection. DCB, drug‐coated balloon; EVT, endovascular therapy; IVUS, intravascular ultrasound; LEAD, lower extremity artery disease; POPA, popliteal artery; SFA, superficial femoral artery.

We screened clinical data from the ASIGARU PAD registry, which included consecutive patients with lower extremity artery disease (LEAD) who underwent endovascular therapy (EVT) for femoropopliteal artery lesion between January 2018 and December 2019. The registry is an ongoing, retrospective, multicenter, observational registry of LEAD, and current data include information from five institutions in the Tokai area in Japan. The inclusion and exclusion criteria details were described previously.^12^ A total of 151 consecutive patients and 151 cases were enrolled, and divided into two groups as follows: 65 cases in the Eluvia group and 86 cases in the drug‐coated balloon group. Of the 65 cases in the Eluvia group, patients for whom IVUS images were obtained were evaluated in this study (Figure 1).

### 2.2 Endovascular revascularization and follow‐up protocol

On the basis of the current guidelines and the package insert instructions of the Eluvia,^1^ dual antiplatelet therapy (DAPT) was primarily employed for at least 2 months. In some cases, type and dosage of antithrombotic agents were adjusted according to the physician's discretion. After sheath insertion, intra‐arterial or intravenous heparin bolus was administered as anticoagulation during the procedure in all cases. The heparin dose was left to the investigators' discretion, and activated clotting time was controlled between 250 and 300 s. Eluvia was implanted, covering the entire lesion, and placed in the least plaque burden area possible.

Clinical follow‐up was conducted at baseline, 1, 3, 6, and 12 months. In addition, examination of ankle‐brachial index (ABI) pressure was performed at each visit, and duplex ultrasound (DUS) was performed at 6 and 12 months.

### 2.3 IVUS analysis

At least two experienced physicians reviewed the IVUS data at each institution. Lesion evaluation using IVUS was performed after wire passage and at the end of the procedure. IVUS data were analyzed using serial images containing proximal and distal information of the lesion. The evaluated IVUS parameters were proximal and distal external elastic membrane (EEM) area, lumen area, stent lumen area, landing plaque burden, and stent edge dissection. In addition, the calcification angle, stent lumen eccentricity, and minimum stent area (MSA) of the entire lesion were also assessed. For chronic total occlusion (CTO) lesions, the wire passage route was also investigated.

### 2.4 Study outcomes

The primary outcome was the identification of predictors of recurrence after Eluvia placement, including IVUS parameters at 12 months. The secondary outcome was target lesion recurrence at 12 months.

### 2.5 Definitions

Hypertension was defined as systolic blood pressure ≥130 mmHg or diastolic blood pressure ≥80 mmHg according to the 2017 American College of Cardiology/American Heart Association guidelines for hypertension and/or a history of treatment with antihypertensive medication. Dyslipidemia was defined as low‐density lipoprotein cholesterol ≥140 mg/dL, high‐density lipoprotein cholesterol <40 mg/dL, or triglyceride ≥150 mg/dL, and/or a history of treatment with antidyslipidemic medication. Diabetes mellitus was defined based on medical history, plasma glucose level, or glycosylated hemoglobin level. The severity of frailty was categorized into three groups as 1–3 (well activities), 4–5 (mild frailty), and 6–8 (moderate or severe frailty) based on the Clinical Frailty Scale (CFS).^13^ The clinical severity of LEAD was assessed using the Rutherford classification, which consists of seven grades (0–6). The femoropopliteal lesion severity was assessed using the Trans‐Atlantic Inter‐Society Consensus (TASC II) classification. The angiographic lesion calcification was evaluated using the peripheral artery calcification scoring system (PACSS) consisting of five grades (0–4), and PACSS grade 4 was defined as severe calcification.^14^ The EEM was defined as the area bounded by the leading adventitial edge, and the lumen area was defined as the area bounded by the luminal border.^15^ The stent edge area was measured as the area inside the stent within 2 mm of the stent edge. The MSA was defined as the smallest area bounded by the stent border.^15^ The plaque burden was calculated by dividing the lumen area by the EEM at the stent edge.^16^ The stent edge dissection was evaluated using the coronary artery dissection classification.^17^ The stent lumen eccentricity was calculated using the following formula: Minimum stent diameter)/maximum stent diameter.^15^ The wire passage route was classified based on the IVUS findings: intraplaque, inside the plaque; subintimal, between the plaque and media; and intramedial, between the media and adventitia.^18^ Technical success was defined as successful completion of the procedure with ≤30% residual stenosis. The target lesion recurrence was defined as a peak systolic velocity ratio ≥2.5 by DUS, >50% stenosis by angiography, or an ABI decrease >0.15 or revascularization. Recurrence patterns were categorized into three grades: Tosaka I (focal stenosis), Tosaka II (diffuse stenosis), and Tosaka III (occlusion).^19^

### 2.6 Statistical analysis

Continuous data are presented as mean ± standard deviation. Categorical data are presented as counts (percentages). An unpaired *t* test was used to compare continuous variables, and the *χ* ^2^ test was used for categorical data. Recurrence predictors were detected using the random survival forest (RSF) method, which is an extension of Breiman's random forest method, allowing efficient nonparametric analysis of time‐to‐event data.^20^, ^21^ According to the RSF, variables with variable importance (VIMP) of >0.002 were defined as potentially predictive, and variables with larger VIMP were considered more predictive.^20^ Moreover, the relationship between the variables and primary patency, and the cutoff points of each predictor were assessed using the partial dependence plot (PDP) method.^22^ The PDP can show whether the relationship between the variables and a feature is linear, monotonic, or more complex. We used the SPSS version 23 (SPSS Inc.) and R version 4.1.1 (the R‐project for Statistical Computing [http://www.R-project.org/]) for all statistical analyses. All *p* values were two‐tailed, with a *p* < 0.05 considered statistically significant for all analyses.

## 3 RESULTS

### 3.1 Baseline clinical characteristics

Of the 65 cases, IVUS images were obtained from 54 cases. The baseline clinical characteristics are shown in Table 1. The mean age was 75.1 years, and 70.4% were men. Diabetes mellitus was present in 57.4% of patients, dialysis‐dependent status in 14.8%, and chronic limb‐threatening ischemia (CLTI) in 29.6%. Out of the population, CFS levels 1–3 accounted for 53.7%, CFS levels 4–5 for 27.8%, and CFS levels 6–8 for 18.5%; there were no patients with CFS level 9.

**Table 1:** Baseline clinical characteristics.

	*n* = 54
*Patient characteristics*	
Age, years	75.1 ± 9.4
Male gender, *n* (%)	38 (70.4)
BMI, kg/m^2^	21.5 ± 3.3
Hypertension, *n* (%)	43 (79.6)
Dyslipidemia, *n* (%)	37 (68.5)
Diabetes mellitus, *n* (%)	31 (57.4)
Hemodialysis, *n* (%)	8 (14.8)
Current smoker, *n* (%)	17 (31.5)
Coronary artery disease, *n* (%)	22 (40.7)
Cerebrovascular disease, *n* (%)	17 (31.5)
CFS, *n* (%)	
1–3	15 (18.1)
4–5	26 (31.3)
6–8	42 (50.6)
Rutherford category, *n* (%)	0.56 ± 0.26
2	11 (20.4)
3	27 (50.0)
4	7 (13.0)
5	6 (11.1)
6	3 (5.5)
CLTI, *n* (%)	16 (29.6)
*Medication*	
DAPT, *n* (%)	47 (87.0)
DOAC, *n* (%)	3 (5.6)

*Note*: Continuous data are presented as means ± standard deviation. Categorical data are given as counts (percentage).

Abbreviations: BMI, body mass index; CFS, Clinical Frailty Scale; CLTI, chronic limb‐threatening ischemia; DAPT, dual antiplatelet therapy; DOAC, direct oral anticoagulants.

### 3.2 Lesion and procedural characteristics

The lesion and procedural characteristics are presented in Table 2. The mean total lesion length was 182.5 mm, and 66.7% had CTO. Severe calcification was observed in 20.4% of the cases. All Eluvia were successfully implanted, covering the entire target lesion, and technical success was achieved in all cases. Pre‐ and postdilatation were performed in 98.1% of cases, and the mean total stent length was 207 mm.

**Table 2:** Lesion and procedural characteristics.

	*n* = 54
*Lesion characteristics*	
Lesion length, mm	182.5 ± 91.3
CTO, *n* (%)	36 (66.7)
Severe calcification (PACSS 4), *n* (%)	11 (20.4)
Poor run‐off (less than 1 artery), *n* (%)	15 (27.8)
TASC II grade C or D, *n* (%)	36 (66.7)
*Procedural characteristics*	
Technical success, *n* (%)	54 (100.0)
Predilatation, *n* (%)	53 (98.1)
Preballoon size, mm	4.8 ± 0.8
Predilatation before IVUS, *n* (%)	15 (27.8)
Postdilatation, *n* (%)	53 (98.1)
Postballoon size, mm	5.6 ± 0.7
Total stent length, mm	207.0 ± 110.7
Minimum stent size, mm	6.3 ± 0.5

*Note*: Continuous data are presented as means ± standard deviation. Categorical data are given as counts (percentage).

Abbreviations: CTO, chronic total occlusion; IVUS, intravascular ultrasound; PACSS, peripheral artery calcification scoring system; TASC II, Trans‐Atlantic Inter‐Society Consensus.

### 3.3 IVUS findings

The IVUS findings are presented in Table 3. The mean EEM area was 47.9 mm^2^ in the proximal portion and 34.5 mm^2^ in the distal portion. Calcification occupying more than 270 degrees of the lumen was observed in 29.6% of cases. At the end of treatment, the mean plaque burden was 40.8% in the proximal stent edge, 40.2% in the distal stent edge, and the mean MSA was 16.8 mm^2^. Stent edge dissection was observed in 25.5% of cases, and dissection extending behind the plaque was observed in 5.9%.

**Table 3:** IVUS findings.

	*n* = 54
*Preprocedural findings*	
Proximal EEM area, mm^2^	47.9 ± 17.0
Distal EEM area, mm^2^	34.5 ± 11.8
Proximal lumen area, mm^2^	28.4 ± 12.1
Distal lumen area, mm^2^	20.9 ± 8.2
Calcification angle, *n* (%)	
None	5 (9.3)
<90°	16 (29.6)
<180°	8 (14.8)
<270°	9 (16.7)
≥270°	17 (29.6)
*Postprocedural findings*	
Proximal stent edge area, mm^2^	24.9 ± 8.5
Distal stent edge area, mm^2^	21.0 ± 7.3
Proximal stent plaque burden, %	40.8 ± 11.6
Distal stent plaque burden, %	40.2 ± 10.0
Minimum stent area, mm^2^	16.8 ± 4.8
Axial lumen eccentricity	0.69 ± 0.12
Wire passage route for CTO lesions, *n* (%)	
Intraplaque	25 (46.3)
Subintimal	10 (18.5)
Intramedial	1 (1.9)
IVUS stent edge dissection grade, *n* (%)	
None	38 (74.5)
A	9 (17.6)
B	1 (2.0)
C	2 (3.9)
D	1 (2.0)

*Note*: Continuous data are presented as means ± standard deviation. Categorical data are given as counts (percentage).

Abbreviations: CTO, chronic total occlusion; EEM, external elastic membrane; IVUS, intravascular ultrasound.

### 3.4 Recurrence rate and predictors of recurrence

**Figure 2:**
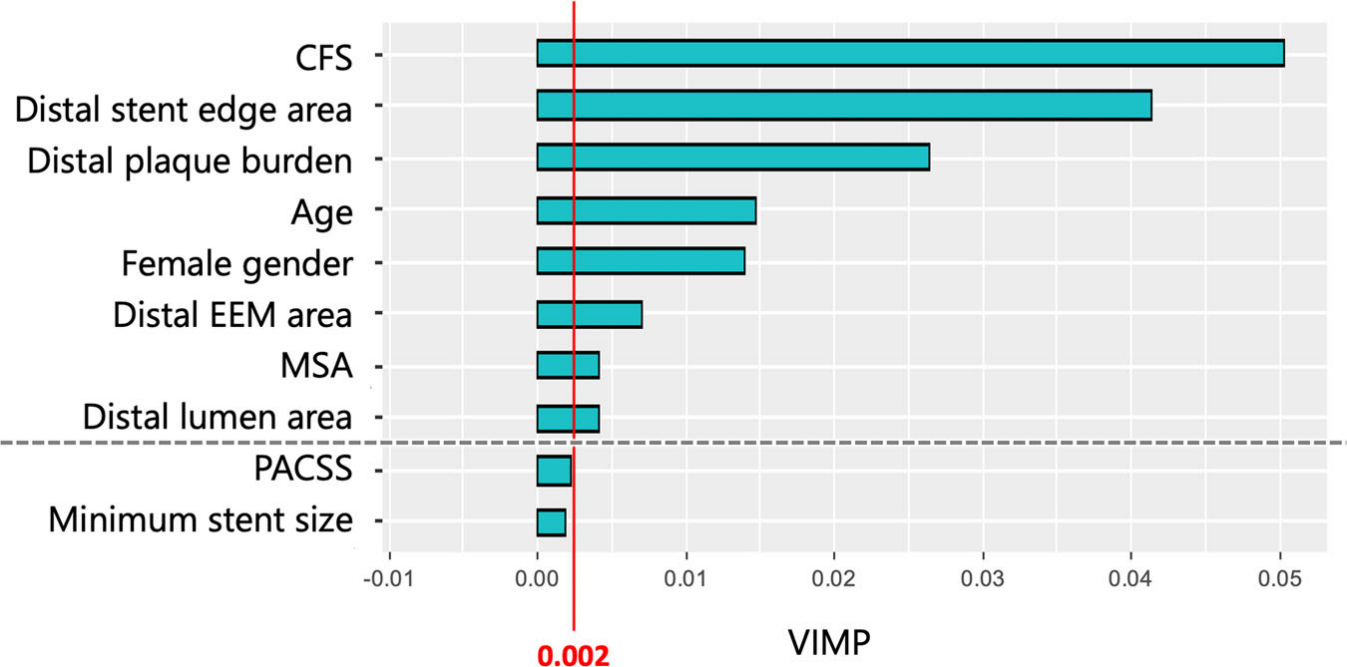
Random Survival Forest analysis of the recurrence predictors. Variables with a VIMP > 0.002 are defined as potentially predictive, and variables with a larger VIMP are considered more predictive. The red line represents a VIMP value of 0.002. The RSF presents eight predictive variables: CFS, distal stent edge area, distal plaque burden, age, sex, distal EEM area, MSA, and distal lumen area. A dotted line indicates the boundaries between significant and nonsignificant factors. CFS, Clinical Frailty Scale; EEM, external elastic membrane; MSA, minimum stent area; PACSS, peripheral artery calcification scoring system; RSF, random survival forest; VIMP, variable importance.

**Figure 3:**
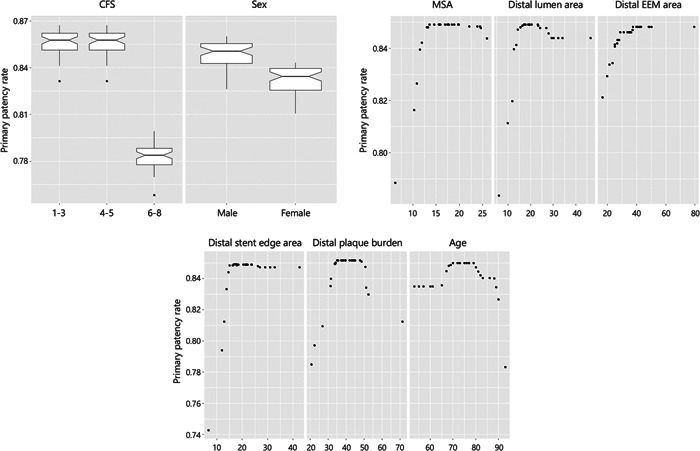
Partial dependence plot results. The upper and lower lines of the boxes represent the 75th and 25th percentiles, respectively. The black vertical lines represent the 90th and 10th percentiles. The dotted curves represent smooth approximate lines for the continuous variables. PDP shows that frailty (CFS ≥ 6), smaller distal stent edge area, higher and lower distal plaque burden, older and younger age, female sex, smaller distal EEM area, smaller MSA, and smaller and larger distal lumen area predict recurrence after Eluvia placement within 12 months. CFS, Clinical Frailty Scale; EEM, external elastic membrane; MSA, minimum stent area; PDP, partial dependence plot.

The clinical results are presented in Table 4. Of the 54 cases, 7 (13.0%) cases of recurrence were observed within 12 months. Two cases had focal stenosis (Tosaka I), four cases had total occlusion (Tosaka III), and one case was unknown because an angiographic examination was not performed. The characteristics of each recurrence case are summarized in the case series (Table 5). The RSF presented eight predictive variables of recurrence (Figure 2): CFS, distal stent edge area, distal plaque burden, age, sex, distal EEM area, MSA, and distal lumen area. Furthermore, PDP showed that frailty (CFS ≥ 6), smaller distal stent edge area, higher and lower distal plaque burden, older and younger age, female sex, smaller distal EEM area, smaller MSA, and smaller and larger distal lumen area predicted recurrence after Eluvia placement within 12 months (Figure 3). In contrast, diabetes mellitus, dialysis‐dependent status, CLTI, CTO, the severity of calcification, lesion length, and stent edge dissection were not significant predictors of recurrence after Eluvia placement.

**Table 4:** Clinical results.

	*n* = 54
Recurrence, *n* (%)	7 (13.0)
Tosaka grade, *n* (%)	
I	2 (28.6)
II	0 (0.0)
III	4 (57.1)
Unknown	1 (14.3)

*Note*: Continuous data are presented as means ± standard deviation. Categorical data are given as counts (percentage).

**Table 5:** Recurrent case series.

	Tosaka grade	Patency term, days	Age, yrs	Sex	Frailty (CFS 6–8)	Diabetes mellitus	Hemodialysis	Indication	Lesion length, mm	Number of run‐off	PACSS	CTO	Proximal EEM area, mm^2^	Distal EEM area, mm^2^	Proximal lumen area, mm^2^	Distal lumen area, mm^2^	Calcification angle	Proximal stent edge area, mm^2^	Distal stent edge area, mm^2^	Proximal plaque burden, %	Distal plaque burden, %	MSA, mm^2^	Stent lumen eccentricity	IVUS stent edge dissection grade
Case 1	III	17	85	Female	Yes	Yes	No	CLTI	300	2	1	Yes	64.9	37.1	36.4	29.3	≥270°	33.7	26.8	44	20.9	24.9	0.32	D
Case 2	I	34	64	Female	No	Yes	No	IC	180	2	4	No	26.8	20.6	12.9	5.9	≥270°	12.4	6.7	51.9	71.4	6.2	0.42	A
Case 3	III	2	91	Female	No	No	No	IC	60	3	1	No	33.3	28.6	19.6	14	<270°	19.3	12.9	41.1	51	10.5	0.28	None
Case 4	I	95	93	Male	Yes	No	Yes	CLTI	73	1	1	No	29.3	26.7	18.6	11	<90°	15.7	14.3	36.5	58.8	11.9	0.19	None
Case 5	III	45	65	Female	Yes	Yes	Yes	CLTI	311.4	1	4	No	39.4	17.7	24.1	11.9	≥270°	23.9	11.6	38.8	32.8	10.8	0.12	None
Case 6	III	341	64	Male	No	No	No	IC	309.4	3	3	Yes	56.6	36	47.7	28	<180°	45.6	26.5	15.7	22.2	22.1	0.13	None
Case 7	Unknown	220	82	Female	Yes	Yes	No	CLTI	205.6	3	1	Yes	59.4	23.2	39.2	17	<90°	26.5	17.8	34	26.7	15.7	0.28	None

Abbreviations: CFS, clinical frailty scale; CLTI, chronic limb‐threatening ischemia; CTO, chronic total occlusion; EEM, external elastic membrane; IC, intermittent claudication; IVUS, intravascular ultrasound; MSA, minimum stent area; PACSS, peripheral artery calcification scoring system.

## 4 DISCUSSION

Eluvia has generally been available in Japan since 2019 and is associated with good clinical performance. The IMPERIAL trial reported that Eluvia presented significantly better clinical results than polymer‐free DES, with 90.9% of primary patency rate and 98.2% of freedom from clinically‐driven TLR rate at 12 months.^9^ In a clinical setting, Eluvia also showed favorable primary patency rates of 87% in the Munster all‐comer registry (lesion length 200 mm, CTO 79%, and moderate or severe calcification 42%), and 84% in the DESAFINADO registry (lesion length 193 mm, CTO 48%, and severe calcification 52%) at 12 months.^10^, ^11^ In the Munster all‐comer registry, predictors of patency loss and TLR were examined, and no predictor of patency loss was detected, whereas distal popliteal artery lesions significantly predicted TLR.^23^ The subgroup analysis in the DESAFINADO registry showed that the total lesion coverage therapy with Eluvia had a better primary patency rate than hybrid therapies with other devices.^11^ However, these studies did not investigate the IVUS findings. Previous reports regarding BMS and polymer‐free DES have presented the importance of IVUS findings for femoropopliteal EVT, and lower plaque burden, smaller distal EEM area, smaller MSA, smaller distal lumen cross‐sectional area, and axial symmetry index were reported to be predictors of recurrence.^8^, ^16^, ^24^ In the IVORY registry, the predictors of recurrence based on IVUS findings were studied in a group of patients, including various femoropopliteal devices.^25^ However, only 1.3% of the patients were implanted with Eluvia in this study, which does not reflect the results of Eluvia placement in a clinical setting. This is the first study to identify predictors of recurrence after Eluvia placement while including IVUS findings.

In this study, eight predictors of recurrence after Eluvia placement were identified. Of these, three were patient characteristics, and five were IVUS findings. A unique aspect of this study was that the IVUS parameters correlated more strongly with recurrence than did lesion characteristics that could be identified by angiography, such as lesion length, CTO, and PACSS grade. This result is supported by the fact that the Munster all‐comer registry did not identify angiographic lesion characteristics as a recurrence factor.^23^

Next, we considered individual factors. Female sex has been reported to be a recurrent factor in previous devices, and the results of our study were similar.^26^, ^27^ Frailty is most often defined as an aging‐related syndrome of physiological decline, characterized by marked vulnerability to adverse health outcomes. The progression of frailty has been reported to affect the outcomes of various diseases.^28^, ^29^ It has been suggested that one of the pathological conditions of frailty is a decrease in the “physiological reserve”, and poor microvasculature of lower extremity and disorder of angiogenesis due to muscle reduction maybe the essence of the cause of the increased recurrence rates.^30^, ^31^ Our previous study reported that the recurrence rate of the femoropopliteal lesions after EVT was higher in frail patients than in nonfrail patients, and the results of this study were also similar.^32^ Concerning age, a strong correlation was found with increasing age and a weak correlation with decreasing age. We hypothesized that elderly patients generally tend to have poor backgrounds, such as various diseases and frailty, leading to higher a recurrence rate. Younger patients with LEAD also tend to have several risk factors. The status of antithrombotic therapy is an important factor in considering patency loss after treatment with Eluvia placement. A previous study has reported that DAPT was able to decrease the TLR rate at 6 months; however, the efficacy did not last up to 12 months.^33^ In our study, DAPT was not a significant predictor for recurrence of superficial femoral and proximal popliteal arteries lesions, and the result was consistent with the previous report. These recurrence predictors of the patient characteristics were consistent with our main report.^12^ Patients with recurrence predictors such as female, severe frailty, and elderly and younger age may be better treated by appropriate devices other than Eluvia. Distal EEM area, distal lumen area, distal stent edge area, distal plaque burden, and MSA were identified as predictors of recurrence related to the IVUS findings. Although the distal lumen area and distal plaque burden showed bilateral correlation (smaller and larger, lower and higher, respectively), most cases with larger distal lumen area or lower distal plaque burden corresponded to multiple patient characteristics predictors as shown in the case series (Table 5). Small distal vessel factors were consistently related to recurrence predictors. Our study revealed that small vessels and high plaque burden were predictors of recurrence in polymer‐coated DES, as well as in BMS and polymer‐free DES.^8^, ^16^ Small vessels tend to lead to small MSA, and a high plaque burden tends to lead to a small stent edge area. Therefore, acquiring a substantial stent lumen area may be a challenge for Eluvia placement.

The following conclusions can be drawn from our study results: to maintain patency of Eluvia in daily clinical practice, it is important to (1) assess vascular information using IVUS, (2) implant in a distal plaque‐free area, and (3) acquire sufficient stent lumen as much as possible. It may be better to avoid implanting Eluvia in lesions where these conditions are difficult to meet. Further research is required to elucidate these speculations in the future.

### 4.1 Limitations

This study has several limitations. First, this was a nonrandomized, retrospective study with a small number of cases. Second, selection bias may have occurred because the IVUS data were unavailable for all patients enrolled in this study. Third, a third‐party organization, such as a core laboratory, did not assess the angiographic and IVUS data. Fourth, only Japanese patients were enrolled in this study, and studies on populations of other races might have shown different results.

## 5 CONCLUSION

CFS, distal stent edge area, distal plaque burden, age, sex, distal EEM area, MSA, and distal lumen area were significant predictors of recurrence after Eluvia placement.

## AUTHOR CONTRIBUTIONS

**Takehiro Yamada**: Conceptualization; project administration; writing—original draft. **Takahiro Tokuda**: Investigation; methodology; writing—review and editing. **Naoki Yoshioka**: Data curation; formal analysis; validation. **Akio Koyama**: Funding acquisition; resources. **Ryusuke Nishikawa**: Software; visualization. **Kiyotaka Shimamura**: Validation. **Takuma Aoyama**: Supervision. All authors have read and approved the final version of the manuscript. Takehiro Yamada had full access to all of the data in this study and takes complete responsibility for the integrity of the data and the accuracy of the data analysis.

## CONFLICT OF INTEREST STATEMENT

The authors declare no conflict of interest.

## ETHICS STATEMENT

This study was conducted following the Declaration of Helsinki and was approved by the ethical committee of each institution (ID: 2020‐036). Written informed consent was obtained from each patient or their relative before the index procedure.

## TRANSPARENCY STATEMENT

The lead author Takehiro Yamada affirms that this manuscript is an honest, accurate, and transparent account of the study being reported; that no important aspects of the study have been omitted; and that any discrepancies from the study as planned (and, if relevant, registered) have been explained.

## Data Availability

The data sets used and/or analyzed during the current study are available from the corresponding author upon reasonable request.
